# Crop rotation significantly influences the composition of soil, rhizosphere, and root microbiota in canola (*Brassica napus* L.)

**DOI:** 10.1186/s40793-023-00495-9

**Published:** 2023-05-09

**Authors:** Jennifer R. Town, Tim Dumonceaux, Breanne Tidemann, Bobbi L. Helgason

**Affiliations:** 1 Agriculture and Agri-Food Canada, Saskatoon Research and Development Centre, Saskatoon, SK Canada; 2 Agriculture and Agri-Food Canada, Lacombe Research and Development Centre, Lacombe, AB Canada; 3 Department of Soil Science, University of Saskatchewan, Saskatoon, SK Canada

**Keywords:** *Olpidium*, Microbial diversity, Crop rotation, Root exudates, Soil nutrients

## Abstract

**Background:**

Crop rotation is an agronomic practice that is known to enhance productivity and yield, and decrease pest and disease pressure. Economic and other factors have increased the frequency of certain crops, including canola, with unknown effects on the below ground microbial communities that impact plant health and performance. This study investigated the effect of 12 years of crop rotation including canola-wheat; canola-pea-barley; and unrotated canola across three geographic sites in Western Canada with diverse soil types and environmental conditions. To provide data on mature, established crop rotation strategies, root exudate profiles, soil nutrient fluxes, and bacterial and fungal microbial community profiles were determined at the flowering stage in the final two (canola) years of the 12-year rotations.

**Results:**

After 12 years of rotation, nutrient fluxes were affected in the soil in an inconsistent manner, with K, NO_3_, Mg, Ca, P, and Fe fluxes variably impacted by rotation depending on the year and site of sampling. As expected, rotation positively influenced yield and oil content, and decreased disease pressure from *Leptosphaeria* and *Alternaria*. In two of the three sites, root exudate profiles were significantly influenced by crop rotation. Bacterial soil, root, and rhizosphere communities were less impacted by crop rotation than the fungal communities. Fungal sequences that were associated with specific rotation strategies were identified in the bulk soil, and included known fungal pathogens in the canola-only strategy. Two closely related fungal sequences identified as *Olpidium brassicae* were extremely abundant at all sites in both years. One of these sequences was observed uniquely at a single site and was significantly associated with monocropped canola; moreover, its abundance correlated negatively with yield in both years.

**Conclusions:**

Long-term canola monoculture affected root exudate profiles and soil nutrient fluxes differently in the three geographic locations. Bacterial communities were less impacted by rotation compared to the fungal communities, which consistently exhibited changes in composition in all ecological niches at all sites, in both years. Fungal sequences identified as *O. brassicae* were highly abundant at all sites, one of which was strongly associated with canola monoculture. Soil management decisions should include consideration of the effects on the microbial ecosystems associated with the plants in order to inform best management practices.

**Supplementary Information:**

The online version contains supplementary material available at 10.1186/s40793-023-00495-9.

## Background

Canola (*Brassica napus* L.) is an important crop for Canadian producers and both the total acreage and frequency of canola on individual farms have increased significantly in the past 20 years [1]. As a result, continuous or 1 in 2-year canola rotations have become increasingly common. It is important to examine the impacts of canola rotation length to support best management recommendations and mitigate agronomic risk with respect to yield and pest management [2].

Diverse crop rotations comprise an important facet of sustainable, resilient production systems aiding in disease suppression, nutrient cycling and risk mitigation [3]. While the importance of crop rotation for reducing pathogen host-crop incidence is acknowledged [4] less is known about the indirect impacts of canola frequency on the broader soil and plant microbiome and how this subsequently affects soil nutrient cycling and uptake. Compared to monoculture, diverse rotations have higher levels of nutrient cycling [5], increases in bioavailable nitrogen, soil organic matter, and available potassium [6], and appear to have broader soil microbial metabolic capabilities, perhaps owing to a greater diversity of residue input types [7, 8]. Canola is known to have diverse and abundant root and rhizosphere microbiomes [9–11]. This microbiome has many important roles in plant health including nutrient uptake, resistance to pests and other stresses and for maintaining soil fertility [12]. Canola tissues have a characteristic biochemical composition (e.g. glucosinolates) that have a variety of roles including allelopathic functions [13] and inhibition of microbial nitrification [14].Therefore, planting fields to canola is expected to have strong short term impacts on soil microbial community structure and function as well as nutrient availability [15]. Whether these effects are temporary [16] or longer lasting may depend on the frequency of canola cropping, since the influence of potentially persistent allelopathic compounds on soil biota is unknown. *Brassica* spp. are among the minority of plants that do not associate with arbuscular mycorrhizal fungi (AMF), ubiquitous symbiotic soil fungi that have important roles in water and nutrient (especially P) acquisition by many other plants [17]. Non-host crops decrease the AMF inoculum potential in the soil and mycorrhizal colonization of subsequent host crops (e.g. cereals) and may decrease translocation of fresh plant C to soil microbes [18–20]. Because canola is not an AMF host crop, it may have other means of acquiring P such as direct excretion of P solubilizing organic acids as reflected in the root exudates. Soil monocropped to maize plants featured decreased P and Mg bioavailability, and the plants themselves had a strong effect on the composition of the soil microbial communities, presumably through interactions of the root/rhizosphere with the soil microbiota [21]. Root exudates aid in plant nutrient acquisition and also support increased abundance and activity of soil microorganisms. Pioneering work on root exudates and mycorrhizal fungal enzymes showed that that plant- and fungal-derived compounds are involved in the turnover of mineral nutrients in *Populus* spp. [22]. In addition, the pattern of the production of root-derived phytochemicals was determined to follow developmental cues and the root exudates produced by the plant were correlated to the metabolic capacity of the soil microbiota in *Arabidopsis* grown in natural soils [23]. The composition of the root-associated microbiota was determined to be selected by the plant at specific stages of Arabidopsis development through the production of root exudates [24]. As a result of this and other work, plant root exudates are now thought to be a primary mechanism through which plants manipulate and shape their microbiomes [25]. Together, these characteristics can act as soil microbiome disruptors, with greater potential benefits and detriments when canola is grown with high frequency.

This study encompasses the final two years of a long-term rotation study to assess the long term (> 10 years) adaptive differences in soil microbiome characteristics for both glufosinate-resistant and glyphosate-resistant canola grown in continuous canola (C-C), canola-wheat (C-W) and canola-pea-barley (C-P-B) rotations. We purposefully chose the final two years of this rotation study to determine the long-term effects of rotation, excluding the potentially confounding temporal effects of crop rotation establishment on soil microbial community profiles. These canola-intensive vs. diverse rotations were examined at three sites on the Canadian prairies by simultaneously measuring nutrient fluxes, changes in soil, rhizosphere and root bacterial and fungal community structures and the secretion of root exudates. We hypothesized that less diverse rotations would result in (1) reduced soil N, P and K fluxes, (2) increased abundance of organic acids secreted from roots, and (3) a reduction in the bacterial and fungal diversity as well as compositional changes in the soil, rhizosphere and root. By examining multiple indicators of plant and soil health, we identified specific agronomic consequences of low diversity canola rotations.

## Methods

### Sample collection

Experimental fields were located at three sites on the Canadian prairies to capture regional differences in climate and soil type. Sites near Swift Current (lat 50.28, long − 107.76) and Scott (lat 50.15, long − 106.58) in Saskatchewan are in the Brown and Dark Brown soil zones, respectively, while the samples taken near Lacombe, Alberta (lat 52.45, long − 113.75) are from the Black soil zone. Mean annual precipitation was 345 mm at Swift Current, 348 mm at Scott and 451 mm at Lacombe (ECCC, ClimateData.ca). As part of a complete randomized block design, four replicate plots at each site were sown with both InVigor® L241C and Roundup Ready® 75–42 canola cultivars in monoculture, canola-wheat or canola-pea-barley rotations. The field study was set up as an all phases rotation ensuring a canola phase was grown for each rotation in every year. Bulk soil, rhizosphere and root samples were sampled in the canola phase of all 3 rotations in both 2018 and 2019, representing the 11th and 12th years of the long-term experiment. At peak flowering, plants from each row (3–4 plants total per plot) were harvested along with the surrounding 1–1.5 kg of soil using a hand trowel, bagged together, and stored on ice until processing, which was completed within 48 h. Bulk soil was mixed and sieved with a 2 mm stainless steel sieve. Plant roots were clipped just below the crown and shaken to discard loosely adhering soil. To collect the strongly-adhering rhizosphere soil, plant roots were immersed in 200 ml of sterile 0.05 M NaCl for 25 min on a rotary shaker at 150 rpm. Rhizosphere soil was collected by centrifugation at 5000×*g* for 15 min [26]. The supernatant from the centrifugation that was used to collect the rhizosphere soil contained the root exudates and was frozen at − 20 °C until analysis (see below). Root samples were then rinsed with additional 0.05M NaCl and cut into small pieces using a sterile scalpel. Aliquots of the sieved bulk soil, rhizosphere soil and root tissue were stored at − 20 °C prior to DNA extraction.

### Agronomic indicators

*Alternaria* blackspot was assessed by collecting 50 green pods per plot at or prior to swathing. Incidence was determined by the number of pods affected with *Alternaria* out of those assessed per plot. After swathing, 50 canola stem bases/roots were collected from each plot, gently washed and dried, and stored in sealed plastic bags at − 20 °C until processed. Incidence was expressed as the percentage of canola stems that exhibited symptoms of blackleg. Plot grain yield was measured using a plot combine harvester outfitted with a sample weigh system. Samples were cleaned and true yield recorded. An approximately 1 kg subsample of the plot yield was taken from the combine and cleaned to be free of weeds prior to quality analysis. A subsample from each cleaned plot sample was taken and seed oil and protein concentrations were determined using a near-infrared reflectance spectrophotometer, as is recommended by the Canadian Grain Commission (https://www.grainscanada.gc.ca/en/grain-research/export-quality/oilseeds/methods-tests.html). Moisture was measured on each sample using a moisture meter and values adjusted to all be reported at the same 8.5% moisture basis.

### Root exudate analysis

The supernatant containing the root exudates was filtered through Whatman No. 42 paper, pH adjusted to 6–7, and concentrated using a vacuum manifold and Strata-X-AW 33 μm Polymeric Weak Anion-Exchange tubes (Phenomenex, USA). Each Strata column was activated with 6 mL of methanol followed by 6 mL of Milli-Q water. Samples (approximately 220 mL) were added to the columns and the vacuum was applied to pull the liquid through the columns and bind the organic acids, with a rate of application such that individual drops came off of the column at approximately 1 drop per second. After sample application, the columns were washed with 2 × 5 mL of 25 mM ammonium acetate (pH 6–7) and the vacuum was applied until all liquid had entered the column and it appeared dry. Organic acids were subsequently eluted from the columns using 2 × 5 mL of 5% ammonium hydroxide dissolved in methanol. The samples were evaporated until dry using a Labconoco RapidVap™ N_2_ Evaporation System (Thermo Fisher Scientific, MA, USA), resuspended in 5 mL of 10 mM KCl, and analyzed using ion chromatography (IC; DIONEX ICS-2000 employing suppressed conductivity detection with an AS18 column; Thermo Fisher Scientific, ON, Canada). Organic acid elution times were compared to standards for formate, malate, oxalate, succinate, and tartrate [27].

### Soil nutrient fluxes

Soil nutrient fluxes were measured using Plant Root Simulator (PRS) Probes® (Western Ag, Saskatoon, SK, Canada) positioned at 3 locations in one row, 2 m apart from each other, within each plot at an approximate depth of 6″. Anion and cation probes were spaced 4″ apart and positioned approximately 4″ from the seed row and 2″ from the fertilizer band. PRS probes® were initially placed 4 weeks prior to sample collection and exchanged after two, four and six weeks from the initial placement (i.e., every two weeks). Anion and cation probes were collected and processed by Western Ag at times corresponding to the period 2 weeks prior to sample collection, at the time of sample collection, and two weeks after sample collection. At collection, all probes were rinsed with deionized water to remove all soil particles and stored in a sealed plastic bag at 4 °C prior to analysis. Adsorbed nutrients were quantified by transferring to sealed plastic bags containing 17.5 mL of 0.5 M HCl and eluted for one hour. Inorganic N (NH_4_ and NO_3_) in the eluent was then determined colorimetrically using automated flow injection analysis with a Skalar San++ Analyzer (Skalar Inc., Netherlands). The remaining nutrients (P, K, S, Ca, Mg, Al, Fe, Mn, Cu, Zn, and B) were measured using inductively coupled plasma (ICP) spectrometry (Optima ICP-OES 8300, PerkinElmer Inc., USA). All standards and controls are prepared in a 0.5M HCl matrix equivalent to that of the samples.

### DNA sequencing

Total genomic DNA was extracted from 250 mg of the soil and rhizosphere samples using the DNeasy PowerSoil extraction kit (Qiagen, Hilden, Germany) after homogenizing samples using the Qiagen vortex adapter and vortexing horizontally for 10 min. Total genomic DNA was extracted from 50 mg of the root samples using the DNeasy Plant kit (Qiagen, Hilden, Germany) after homogenizing samples in extraction buffer using a mortar and pestle. The V3–V4 region of the 16S rRNA universal target was amplified over 30 cycles (95 °C 30 s, 55 °C 30 s, 72 °C 30 s) using 342F (CTACGGGGGGCAGCAG) and 806R (GACTACHVGGGTWTCTAAT) primers [28]. To profile the fungal community, the ITS1 region was amplified over 30 cycles using ITS1f (CTTGGTCATTTAGAGGAAGTAA) and ITS2 (GCTGCGTTCTTCATCGATGC) primers [29]. Each 25 µL amplification reaction contained 1X PCR Buffer (Thermo Fisher Scientific, MA, USA), 2.5 mM MgCl_2_, 0.5 mM dNTPs, 0.4 µM of each primer and 1U of Platinum Taq polymerase (Thermo Fisher Scientific, MA, USA). For amplifications from root tissue, peptide nucleic acid (PNA) clamps targeting chloroplast and mitochondrial sequences [30] were heated to 60 °C for 10 min and added to the PCR mix to a final concentration of 1 µM each. Both extraction and amplification negative controls containing only buffers and water were processed and sequenced along with experimental samples. Indexed 16S and ITS amplicons were sequenced using 300PE and 250PE cycles of Illumina Miseq Chemistry (Illumina, San Diego, CA, USA).

DNA sequencing reads were processed with Cutadapt (v.2.8) [31] to remove amplification primer sequences and trim bases with a quality score < Q30. Forward and reverse sequencing reads were then merged using FLASH2 (v.2.2) [32] and amplicon sequence variants (ASVs) for both 16S and ITS targets were identified using the q2-DADA2 [33] plugin for QIIME2 (v.2020.2) [34]. Taxonomic classification of the 16S and ITS ASVs was done by Naïve Bayes Classification using the q2-classifier in QIIME2 and the SILVA (released 2019/12/1) [35] and Unite (released 2020/02/04) [36] reference sets respectively. All ASVs less than 350 bp (16S) and 100 bp (ITS) were considered to be less than full length and removed. Additionally, all ASVs occurring in fewer than 5 samples as well as any 16S ASVs that were classified as mitochondrial or chloroplast sequences were removed.

### Quantitative PCR

qPCR was used to validate differential read abundance profiles for taxa identified through sequencing, and provide tools to measure the presence and abundance of particular ASV in other soil samples. Accordingly, a multiplex quantitative PCR assay targeting nucleotide differences in the ITS1 region was used to enumerate fungal taxa ASVd3f1 and ASVf509 in genomic DNA samples from root, rhizosphere and soil. Primers common to the ITS1 region of both sequences as well as ASV-specific Taqman probes were designed using Primer3. Each multiplex reaction contained 1X Ssofast Universal Probes Master Mix (Biorad, CA, USA), 0.3 µM of each primer, 0.2 µM of each TaqMan probe and 2 µL of genomic DNA. Both d3f1 and f509 targets were simultaneously amplified in a multiplex reaction over 40 cycles (95 °C 30 s, 55 °C 30 s, 72 °C 30 s). Primer and probe sequences are provided in Additional file 1. Prior to application to soil samples, the qPCR probes were evaluated to ensure that no cross-reactivity was observed using each of the cloned *O. brassicae* ITS sequences. Each probe detected only the targeted ASV to the exclusion of the other, closely related target ASV (Additional file 1). Plasmids containing the ITS1 sequence for each strain were used as standards to calculate the number of ITS gene copies per gram of soil or root tissue. The relationship between the abundance of *O. brassicae* and yield was tested using Spearman’s rank-order correlation (ρ).

### Statistical methods

Soil nutrient fluxes, root exudates and crop agronomic performance indicators were analysed by MANOVA Benjamini–Hochberg false discovery rate correction and post-hoc significance evaluated with Tukey tests using the ‘stats’ and ‘multcomp’ packages in R. Principal components analysis (PCA) was done using the FactoMineR package [37]. Microbial community diversity statistics including Bray–Curtis dissimilarity and Shannon diversity were calculated after rarefaction to the smallest library size (5000 reads per sample) with the ‘phyloseq’ [38] and ‘vegan’ [39] packages in R. Differences in alpha and beta diversity were tested for significance using the Kruskal–Wallis test (Benjamini–Hochberg false discovery rate correction) and permutational multivariate analysis of variance (PERMANOVA) respectively. Indicator species analysis was conducted using the ‘indicspecies’ package and ASVs that were significantly associated with canola intensity (specificity ‘A’ and sensitivity ‘B’ ≥ 0.7, *p* value ≤ 0.05) at a each site in at least one sample type across both 2018 and 2019 were retained. Differential abundance analysis was calculated using centred log-ratio transformed abundance data with the ‘ALDEx2’ [40] package in R. Differentially abundant taxa were identified as having an effect size ≥ 1 and a Benjamini–Hochberg adjusted *p* value ≤ 0.05 across samples from both 2018 and 2019.

## Results

### Soil nutrient fluxes

The effect of crop rotation on soil nutrient fluxes was variable depending on the field site and sampling year. Principal component analysis (PCA) of the PRS data showed that at Swift Current, canola intensity was associated with significant differences in soil nutrient flux profiles before and during peak flowering in both the 2018 and 2019 growing seasons (Table 1). At both Scott and Lacombe, differences were observed only at single time points in 2019; before flowering at Scott and during peak flowering at Lacombe (Table 1). Cultivar selection was not associated with significant differences in soil nutrient flux profiles at any site (*p* > 0.05, Additional file 2).

**Table 1:** Principal component analysis (PCA) of soil nutrient flux data measured by Plant Root Simulator probes at two week intervals before, during and after peak flowering at Swift Current, Scott, and Lacombe

	*pseudo-F*	*p* value
Lacombe		
2018		
Before	0.974	0.475
Peak	1.133	0.317
After	0.863	0.602
2019		
Before	1.159	0.284
Peak	**2.300**	**0.007**
After	1.302	0.211
Scott		
2018		
Before	0.741	0.760
Peak	0.808	0.635
After	0.821	0.643
2019		
Before	**1.908**	**0.027**
Peak	0.770	0.735
After	1.048	0.397
Swift Current		
2018		
Before	**2.561**	**0.004**
Peak	**2.811**	**0.005**
After	0.725	0.755
2019		
Before	**1.741**	**0.050**
Peak	**1.657**	**0.050**
After	1.259	0.227

PERMANOVA analysis was used to compare the nutrient flux profiles of the soil under C-C, C-W or C-P-B rotations, and significant differences are highlighted in bold (*p* ≤ 0.05)

**Fig. 1:**
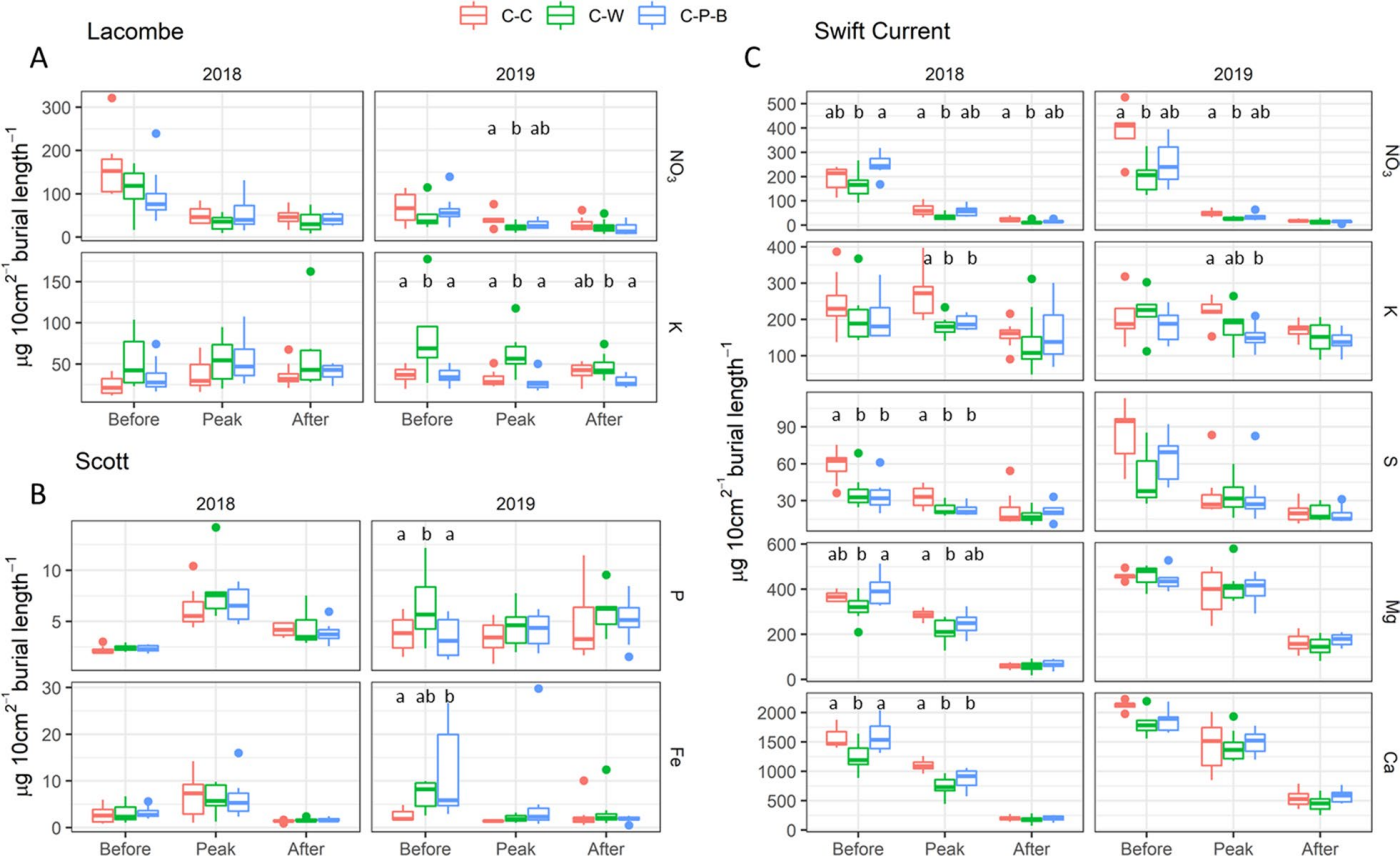
Soil nutrient fluxes measured by Plant Root Simulator probes at two week intervals before, during and after peak flowering at Lacombe (**A**), Scott (**B**) and Swift Current (**C**). The amount of specific nutrients that adsorbed to the probes were measured and compared using MANOVA, with post-hoc Tukey comparison and Benjamini–Hochberg FDR correction. Significant differences are indicated by lowercase letters (*p* ≤ 0.05)

In terms of soil nutrient fluxes for specific macro- and micronutrients, Swift Current saw an increase in K fluxes from continuous canola in both growing seasons, and an increase in soil S in 2018 (Fig. 1). NO_3_ fluxes were higher in the C-P-B rotation in 2018 however in 2019, they were higher in the monocropped canola (Fig. 1). Both Mg and Ca were significantly lower in C-W compared to both monocropped canola and C-P-B in 2018 at the early and peak flowering time points. In 2019 at Scott, diverse rotation was associated with significant differences in P and Fe while at Lacombe, significant differences in NO_3_ and K were associated with rotation (Fig. 1). No significant differences were found between rotations for NH_4_, Mn, B or Al and Cd, Zn, B, Cu and Pb were consistently below the limit of detection (Additional file 2).

### Agronomic performance indicators

Significant differences in soil moisture, crop yield, oil and protein content were restricted to only some sites in some years. At Swift Current in 2019, soil moisture, yield and oil content were all higher in C-P-B canola while protein content was lower. Yield was also significantly higher in C-P-B canola at Lacombe in 2019, but no difference was observed at Scott (Table 2, Additional file 3). There was a general trend of higher oil content in C-P-B canola at both Scott and Lacombe, however the increases were not statistically significant (Table 2, Additional file 3). Disease surveys showed that both the incidence and severity of Blackleg symptoms were significantly higher in C-C canola at all sites, however the average incidence in C-C canola at Swift current was only 21 ± 9.9% across both growing seasons compared to 67 ± 16.0% in Scott and 83 ± 14.7% in Lacombe (Table 3). Symptoms of *Alternaria* black spot on seed pods were detected only at Lacombe in 2019 and were also significantly higher in C-C (9 ± 5.4%) compared to both C-W (5 ± 1.4%) and C-P-B (4 ± 1.3%) (Table 2, Additional file 3).

**Table 2:** Agronomic indicators for canola grown at different rotation intensities at Lacombe, Scott and Swift Current

	2018			2019		
	C-C	C-W	C-P-B	C-C	C-W	C-P-B
Lacombe						
Moisture (%)	36.0 (2.1)	35.3 (3.0)	34.9 (2.9)	33.9 (7.4)	29.9 (2.2)	32.1 (4.7)
Yield (kg/ha)	2952 (838)	3650 (523)	3676 (392)	3151 (269)**A**	3279(586)**A**	3952(421)**B**
Oil (%)*	44.1 (1.5)	45.3 (1.5)	45.5 (1.6)	43.7 (1.3)	43.8 (1.7)	44.5 (1.0)
Protein (%)*	20.6 (0.4)**A**	19.6 (1.0)**B**	19.3 (0.5)**B**	20.7 (0.5)	20.3 (0.4)	20.3 (0.6)
Blackleg (%)	80.8 (16.4)**A**	28.3 (21.4)**B**	28.8 (14.8)**B**	85.3 (13.4)**A**	62.8 (25.4)**AB**	48.3 (27.8)**B**
Alternaria (%)	ND	ND	ND	9.1 (5.4)**A**	4.7 (1.4)**B**	4.3 (1.3)**B**
Scott						
Moisture (%)	19.1 (2.4)	19.9 (2.6)	17.8 (2.6)	17.3 (1.6)	18.4 (2.5)	17.5 (3.4)
Yield (kg/ha)	2642 (382)**A**	2464 (268)**AB**	2252 (165)**B**	2882 (375)	2861 (152)	3086(429)
Oil (%)*	43 (0.9)	44.1 (1.4)	44 (1.7)	45.8 (1.5)	47 (1.6)	46.7 (2.4)
Protein (%)*	21.1 (1.5)	20.2 (2.2)	19.7 (2.3)	19.9 (1.3)	18.7 (2.1)	18.9 (2.6)
Blackleg (%)	58 (12.3)**A**	34.8 (13)**A**	22.5 (10)**B**	76.8 (13.9)**A**	61.3 (15.4)**AB**	28 (26.2)**B**
Alternaria (%)	ND	ND	ND	ND	ND	ND
Swift Current						
Moisture (%)	8.3 (0.6)	9.4 (1.6)	9.8 (2.8)	8.4 (0.8)**A**	9.5 (1.0)**AB**	9.9 (1.3)**B**
Yield (kg/ha)	688 (111)	608 (82)	649 (141)	1872 (105)**A**	2294 (93)**A**	2421 (154)**B**
Oil (%)*	40.1 (1.4)	39.8 (1.3)	40.1 (0.9)	40 (1.9)**A**	42.9 (1.7)**B**	41.9 (2.4)**AB**
Protein (%)*	27.1 (0.6)	27.1 (0.5)	27 (0.5)	24.6 (0.5)**A**	21.3 (0.9)**B**	22.6 (0.8)**C**
Blackleg (%)	16 (7.5)**A**	20 (6.4)**A**	6.5 (2.6)**B**	28.7 (8.2)**A**	15.3 (7.0)**B**	22.8 (6.9)**AB**
Alternaria (%)	ND	ND	ND	ND	ND	ND

*At 8.5% moisture, *ND* not detected

Values were compared using MANOVA analysis with Benjamini–Hochberg false discovery rate correction and significant differences between rotation intensities indicated by bold uppercase letters (*p* ≤ 0.05)

**Table 3:** Quantification of secreted of organic acids (mg L^−1^) from canola roots at Lacombe, Scott and Swift Current under different canola intensities

	Lacombe			Scott			Swift Current		
	C-C	C-W	C-P-B	C-C	C-W	C-P-B	C-C	C-W	C-P-B
Formate	1.86 (0.35)**A**	1.37 (0.24)**B**	1.45 (0.24)**B**	1.62 (0.19)	1.58 (0.18)	1.8 (0.47)	1.74 (0.49)**A**	1.24 (0.34)**B**	1.2 (0.15)**B**
Oxalate	1.72 (0.41)**A**	1.18 (0.25)**B**	1.26 (0.25)**B**	2.28 (0.56)	2.29 (0.88)	2.66 (1.03)	1.72 (0.54)	1.25 (0.36)	1.4 (0.54)
Succinate/malate	2.94 (1.6)	2.48 (1.06)	3.19 (0.69)	3.11 (1.87)	3.33 (1.22)	3.74 (1.29)	1.75 (0.36)	7.64 (9.37)	5.95 (3.14)
Citrate	3.07 (2.15)	1.95 (1.11)	1.71 (1.06)	4.15 (3.87)	2.54 (1.19)	3.54 (1.05)	3.39 (2.03)	11.79 (12.87)	11.24 (6.34)
Tartarate	5.56 (1.11)**A**	3.42 (0.4)**B**	3.79 (0.71)**B**	3.46 (0.65)	3.56 (0.51)	4.11 (0.81)	5.24 (2.58)**A**	5.27 (1.79)**A**	10.33 (5.79)**B**

Differences in the concentration of root exudates were compared using MANOVA analysis with Benjamini–Hochberg FDR correction and significant differences between C-C, C-W or C-P-B rotations indicated by uppercase letters (*p* ≤ 0.05)

### Root exudate secretion

Principal component analysis (PCA) of the root exudate data showed that at Lacombe, canola intensity, but not cultivar selection, was associated with significant differences in overall root exudate composition (PERMANOVA, *F* = 5.024, *p* = 0.002), while there were no significant differences found at either Swift Current or Scott (Additional file 4).

At Swift Current, tartarate was significantly higher in the root exudates from the C-P-B rotation while formate was significantly higher in monocropped canola. At Lacombe, tartarate, formate and oxalate were all significantly higher in monocropped canola compared to either C-W or C-P-B (Table 3). There were no significant differences between rotations observed in samples from Scott.

### Microbial community composition

**Fig. 2:**
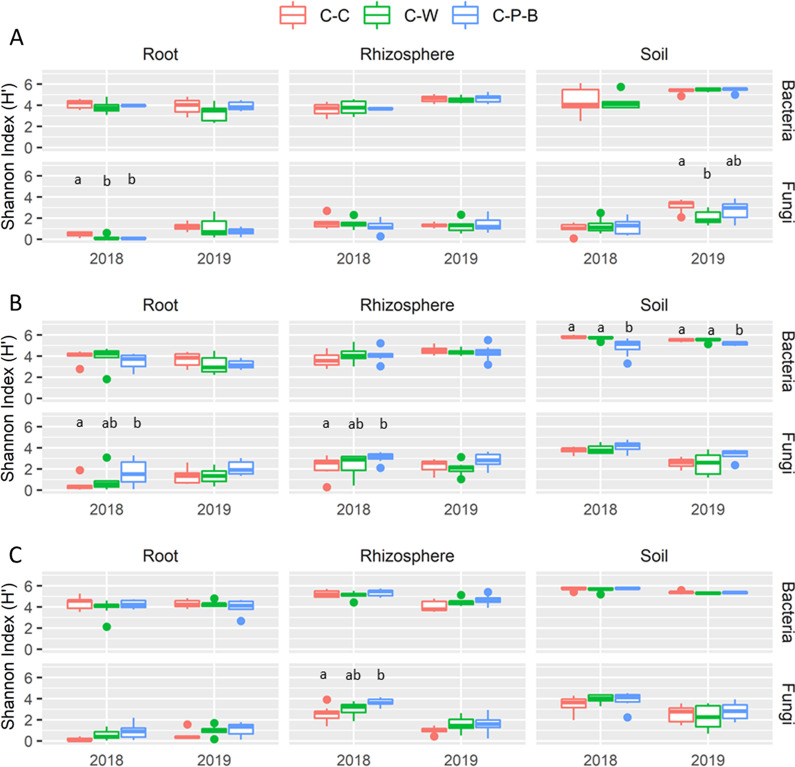
Bacterial and fungal Shannon diversity index for soil, root and rhizosphere communities at Lacombe (**A**), Scott (**B**) and Swift Current (**C**). Differences in microbiome diversity were compared using Kruskal–Wallis test and significant differences indicated with lower case letters (*p* ≤ 0.05)

Alpha diversity analysis showed no consistent effects of rotation on bacterial or fungal diversity across sample types or sites (Fig. 2). At Lacombe and Swift Current, only the bulk soil bacterial community showed a significant decrease in the Shannon Index for C-P-B compared to C-C and C-W. By contrast, the fungal community was significantly more diverse in the C-P-B rhizosphere samples at Swift Current and the rhizosphere and root at Scott, although only in 2018 (Fig. 2).

Beta diversity analysis revealed that the effects of crop rotation on bacterial community composition were variable depending on the year and study site, while fungal community composition was significantly affected across all sites in the soil, rhizosphere and root in both years (Table 4). PERMANOVA analysis of the Bray–Curtis dissimilarity metric also indicated that the effect of crop rotation on the fungal community was greater compared to the bacterial community, especially in samples from the root and rhizosphere, at all sites (Table 4). Generally, cultivar selection was not associated with significant changes in bacterial or fungal community composition with the exception of the bacterial community in root samples from Lacombe, although the effect was small (PERMANOVA, *F* = 1.32, *p* = 0.03) (Additional file 5).

**Table 4:** Effect of canola intensity on the Bray–Curtis dissimilarity index for the bacterial (16S) and fungal (ITS) communities in the soil, root and rhizosphere Swift Current, Scott and Lacombe

	Swift Current			Scott			Lacombe		
	*pseudo-F*	R^2^	*p* value	*pseudo-F*	R^2^	*p* value	*pseudo-F*	R^2^	*p* value
16S Bray–Curtis PERMANOVA									
Soil									
2018	1.195	0.053	0.158	**2.860**	**0.111**	**0.001**	0.864	0.078	0.587
2019	**1.908**	**0.080**	**0.020**	**1.847**	**0.086**	**0.051**	**2.813**	**0.113**	**0.001**
Rhizosphere									
2018	**1.489**	**0.064**	**0.033**	1.218	0.053	0.190	**2.583**	**0.111**	**0.018**
2019	**2.624**	**0.107**	**0.010**	1.050	0.046	0.361	**2.048**	**0.088**	**0.001**
Root									
2018	**1.488**	**0.063**	**0.042**	**2.290**	**0.090**	**0.001**	1.382	0.068	0.087
2019	**2.624**	**0.091**	**0.017**	1.241	0.056	0.140	**2.032**	**0.081**	**0.002**
ITS Bray–Curtis PERMANOVA									
Soil									
2018	**2.236**	**0.092**	**0.001**	**2.571**	**0.101**	**0.008**	**10.331**	**0.323**	**0.002**
2019	**2.794**	**0.115**	**0.031**	**5.330**	**0.200**	**0.001**	**6.266**	**0.224**	**0.001**
Rhizosphere									
2018	**6.522**	**0.227**	**0.001**	**2.692**	**0.111**	**0.043**	**7.965**	**0.271**	**0.001**
2019	**2.710**	**0.114**	**0.057**	**3.239**	**0.135**	**0.022**	**11.515**	**0.325**	**0.001**
Root									
2018	**7.011**	**0.226**	**0.004**	**5.302**	**0.197**	**0.006**	**18.121**	**0.426**	**0.001**
2019	**5.424**	**0.207**	**0.013**	**2.237**	**0.095**	**0.053**	**18.068**	**0.463**	**0.001**

Differences in microbiome composition in the soil, rhizosphere and root under C-C, C-W or C-P-B rotations were compared using PERMANOVA, and significant differences are highlighted in bold (*p* ≤ 0.05)

**Fig. 3:**
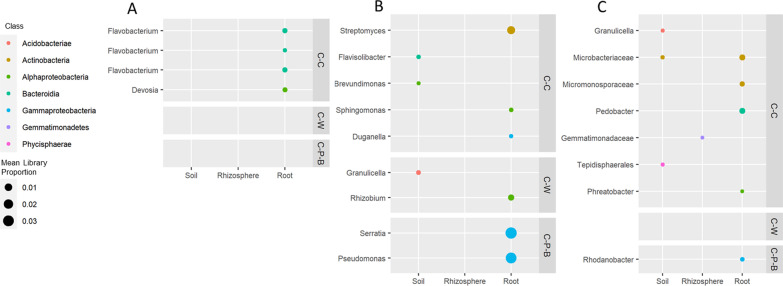
Bacterial 16S indicator ASV uniquely associated with canola from C-C, C-W or C-P-B rotations in the soil, rhizosphere and root Lacombe (**A**), Scott (**B**) and Swift Current (**C**). Sequences were identified using indicator species analysis and retained if both indicator values ‘A’ (specificity) and ‘B’ (sensitivity) were ≥ 0.7 and *p* ≤ 0.05 across both 2018 and 2019 growing seasons

Indicator species analysis identified several ASV sequences that were consistently associated with specific rotation strategies in at least one sample type (bulk soil, rhizosphere or root) in both 2018 and 2019 (Fig. 3, Additional file 6). Across all three sites, there were more fungal ASVs (69) identified as indicator species compared to bacterial ASVs (21). The majority of bacterial indicator ASVs identified were from root samples (15/21) and classified as Actinobacteria (3), Bacteroidota (5) and Proteobacteria (9), however none were consistent across more than one site (Fig. 3, Additional file 6). Roots from C-C samples were associated with multiple *Flavobacterium* spp. at Lacombe and multiple Actinobacteria at Scott and Swift Current including *Streptomyces*, *Microbacteriaceae* and *Micromonosporaceae*. Root samples from the C-P-B rotation at Scott were significantly associated with abundant bacterial ASVs classified as *Serratia* and *Pseudomonas*, comprising a mean of 3.0% and 3.3% of the total sequences respectively (Fig. 3B).

**Fig. 4:**
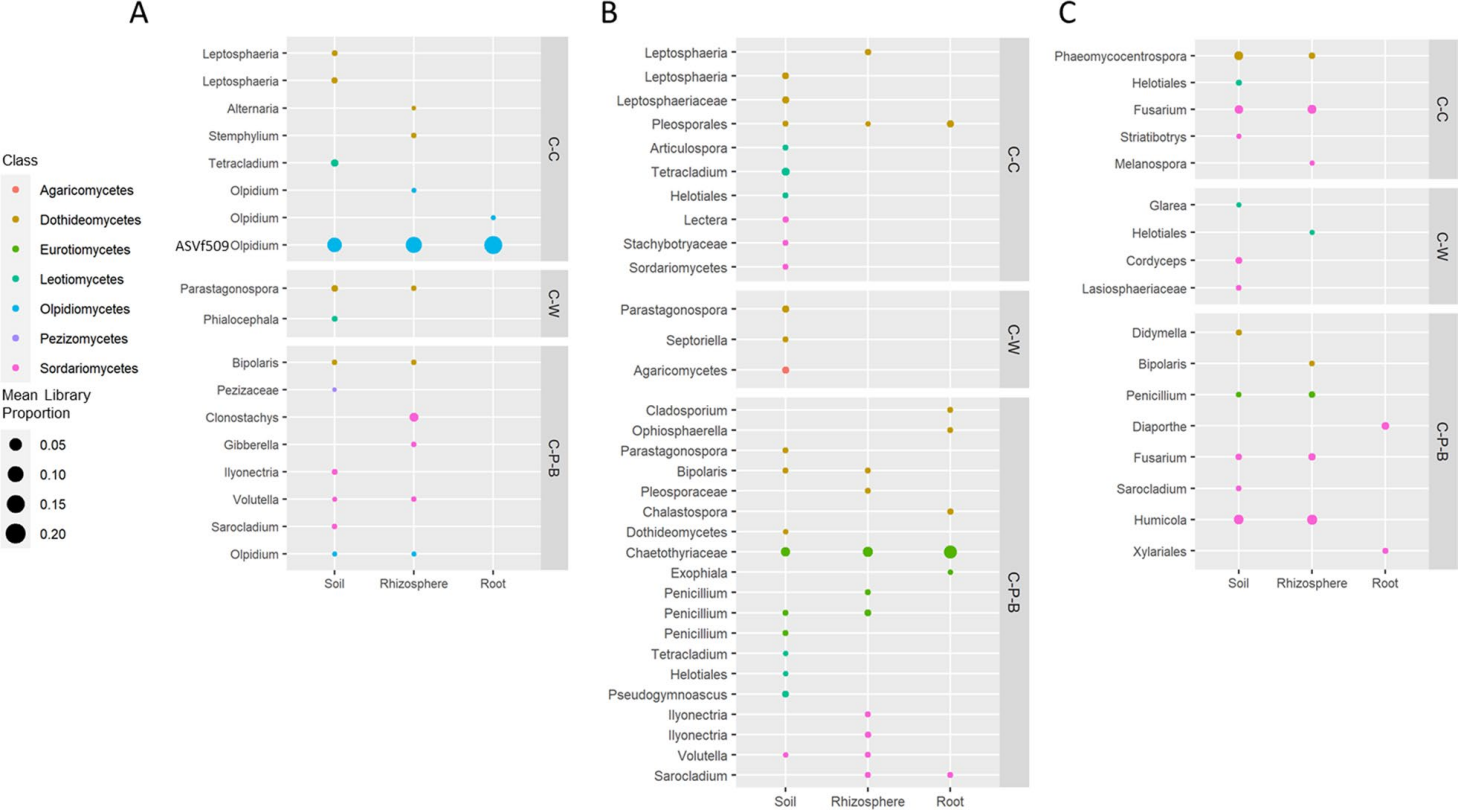
Fungal ITS indicator ASV uniquely associated with canola from C-C, C-W or C-P-B rotations in the soil, rhizosphere and root at Lacombe (**A**), Scott (**B**) and Swift Current (**C**). Sequences were identified using indicator species analysis and retained if both indicator values ‘A’ (specificity) and ‘B’ (sensitivity) were ≥ 0.7 and *p* ≤ 0.05 across both 2018 and 2019 growing seasons

While the majority of bacterial indicator ASVs were root-associated, the majority of fungal indicator ASVs were found in bulk soil samples (52/69). Several indicator ASVs were associated with specific rotations at multiple sites including *Leptosphaeria* and *Tetracladium* with C-C, *Parastagonospora* with C-W and *Penicillium, Volutella, Bipolaris* and *Sarocladium* with C-P-B (Fig. 4). Several of the fungal ASV that were strongly associated with C-C are known phytopathogens, including *Alternaria, Leptosphaeria,* and *Phaeomycocentrospora* (Fig. 4). At Swift Current, *Fusarium* spp. were identified as indicator taxa in the soil and rhizosphere for both C-C and C-P-B however the sequence in C-C was a closer match to *Fusarium domesticum* (96.5%) while the one from C-P-B was a closer match to *Fusarium solani* (96.5%). The majority of indicator ASV associated with C-P-B at Swift Current (5/9) and Lacombe (5/11) were Sordariomycetes, while at Scott indicator taxa also included multiple Leotiomycetes (3/20) and Eurotiomycetes (5/20). One particularly abundant Eurotiomycetes sequence, classified as *Chaetothyriaceae*, was significantly associated with C-P-B in the soil, rhizosphere and root at Scott with corresponding mean sequence abundances of 1.7%, 2.2% and 5.4% respectively.

**Fig. 5:**
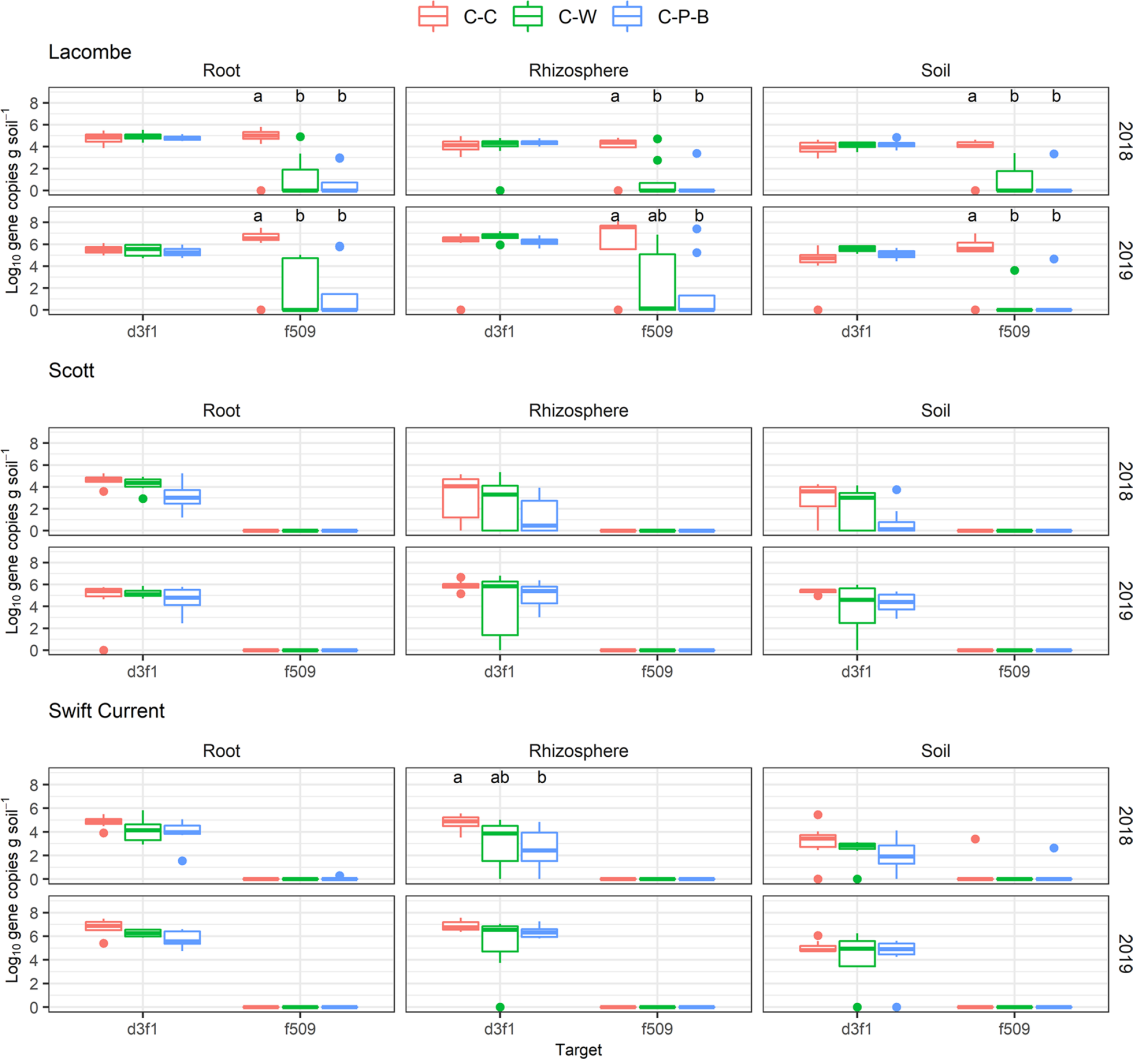
Abundance of *Olpidium brassicae* sequences d3f1 and f509 in root, rhizosphere and soil samples from 2018 and 2019 at Lacombe, Scott and Swift Current. Significance was tested using ANOVA (α = 0.05) and differences indicated with lowercase letters

Two sequences closely related to *Olpidium brassicae* were hyper-abundant in root samples at all three sites, with *Olpidium* spp. accounting for 41–99% of the ITS sequencing libraries (Fig. 4). While samples from Swift Current and Scott were dominated by only one of these sequences (ASVd3f1), samples from Lacombe contained two *O. brassicae* sequences, ASVd3f1 and ASVf509, with ASVf509 significantly associated with soil, rhizosphere and root samples from C-C (Fig. 4A). ASV-specific quantitative PCR analysis confirmed that *O. brassicae* ASVf509 was significantly more abundant in the root, rhizosphere and soil in samples from monocropped compared to rotation canola at Lacombe (Fig. 5), but was only rarely detected in samples from either Scott or Swift Current. The abundance of strain ASVd3f1 in root samples correlated negatively to yield at only Swift Current in 2019 (ρ =  − 0.52, *p* < 0.01) while strain ASVf509 correlated negatively to yield at Lacombe in both 2018 (ρ =  − 0.41, *p* < 0.05) and 2019 (ρ =  − 0.47, *p* < 0.05) (Additional file 7).

Differential abundance analysis using ALDEx2 also identified several fungal Orders that were significantly more abundant (ALDEx2 effect size > 1, *p*.adj ≤ 0.05) in rotation canola in multiple sample types across all sites. In contrast, no differentially abundant bacterial Orders were identified (Additional file 8). Among the differentially abundant fungal Orders, Xylariales were significantly more abundant in the soil, root and rhizosphere of rotation canola at both Scott and Swift Current, but this was not the case at Lacombe. Taxonomic Orders associated with arbuscular mycorrhizal fungi (AMF), Glomerales and Paraglomerales, were also significantly more abundant in soils from rotation canola at both Scott and Lacombe (Additional file 8). The only fungal Orders that were significantly more abundant in continuous canola (C-C) soils were Glomerellales at Scott and Melanosporales at Swift Current.

## Discussion

The effects of rotation on soil moisture, nutrient fluxes and agronomic performance varied depending on the site and year. Swift Current, which was the most arid of the three locations, saw the most benefit from higher diversity rotations including increases in yield, oil content, and soil moisture in 2019. At Lacombe and Scott, where available soil moisture was higher, differences in protein and oil content were not significant, and an increase in yield was only observed in C-P-B canola at Lacombe in 2019. These observations suggest it was more likely soil and environmental factors like precipitation that led to the differences in nutrient fluxes that were observed, as opposed to differences in the amount of available biomass or microbiome composition. The addition of rotation–specific fertilizer each year likely helped to ensure adequate nutrient availability for crop growth, providing evidence that soil-test based fertilizer application is an important tool for managing nutrient supply for short rotations.

Canola frequency effects on bacterial community diversity and composition in the soil, rhizosphere and root were not consistently found at either Scott or Lacombe, a phenomenon also observed by Floc’h et al. [11] comparing rhizosphere communities in C-C, C-W and C-P-B rotations after eight years. At Swift Current, shifts in bacterial community composition were observed in all but one instance, indicating that crop rotation may be most impactful for soil bacteria in this soil zone and under arid conditions.

Crop rotation was associated with a much larger effect on the fungal community of soil, rhizosphere and root with significant changes in composition observed in all sample types at all sites. Kracmarova et al. [41] also observed a more pronounced effect of long-term crop rotation (potato, winter wheat, and spring barley) on soil fungal communities compared to the bacterial microbiota. Rotations of rice and canola were also determined to result in increased soil microbial diversity, and the keystone taxa identified in these rotations were all fungal genera [42]. In the case of canola, the stronger impact of crop rotation on the fungal community may be related to the fact that canola is a non-mycorrhizal crop [43] and thus with increasing canola frequency there will be a selective pressure on these important symbionts. Indeed, the relative abundance of Glomerales and Paraglomerales was lower in C-C soil, a finding consistent with previous research quantifying the effects of canola intensity on AMF diversity in soil [18]. The fungi that were consistently associated with monocropping are known pathogens of canola including *Alternaria*, *Leptosphaeria* and *Phaeomycocentrospora*. Disease assessments confirmed higher incidence of Blackleg symptoms in C-C canola at all sites, a finding in line with previous observations of increased disease pressure in low diversity canola systems [2]. Including wheat or barley in the rotation did increase the relative abundance of known cereal pathogens including *Bipolaris*, *Sarocladium*, *Parastagonospora* and *Volutella*, however rotation canola was also associated with multiple beneficial microorganisms at all sites. Both *Pseudomonas* and *Serratia* found in the roots of C-P-B canola at Scott as well as *Penicillium* in the soil and rhizosphere of C-P-B canola at Scott and Swift Current, have been previously associated with P solubilization in soil and benefits to plant growth [44, 45]. In addition, *Serratia plymuthica* isolated from the rhizosphere of various plant species has been shown to suppress the growth of soil-borne pathogens [46]. Dark septate endophytes such as *Exophiala* and *Chaetothyriaceae*, which were found to be enriched in C-P-B canola at Scott, have been associated with multiple benefits to crop plants including resistance to *Verticillium* wilt, drought tolerance and parasitic activity against nematode eggs [47].

Crop plants exert control over the composition and structure of their microbiome by secreting organic compounds from their roots [48]. Of the three sites, the Lacombe site had the most pronounced effects on fungal community composition in both the rhizosphere and root and was the only location where canola intensity had a significant effect on the root exudate profile. This suggests that field location and associated environmental conditions may have exacerbated the microbiome-related effects of low diversity rotations. Given the unique pattern of abundance of *O. brassicae* ASV f509 in root samples from Lacombe, there exists the possibility of a feedback loop between *O. brassicae* colonization and root exudate secretion with currently unknown resulting effects on plant health. *O. brassicae* is a known endophyte of canola and has been previously found in the roots and rhizosphere of canola across multiple pedoclimatic zones in Canada [10, 12]. While not a known pathogen, *O. brassicae* has been previously associated with short-rotation canola, has been reported to reduce yield at very high infection levels in controlled greenhouse trials [49], and was correlated with yield decline in short rotations in a multi-location field study in the United Kingdom [50]. In our study, the relationship between *O. brassicae* abundance and reduced yield was dependent on both the strain and location. While other members of the genus *Olpidium*, in particular *Olpidium virulentis*, are known vectors for plant viruses [51], widespread reclassification within this genus means the potential of *O. brassicae* to harbour and transmit viruses that may impact canola yield requires further investigation. In this context, it is germane to note that rhizosphere, root, and bulk soil feature very high spatial and temporal diversity in virome composition in *B. napus*, with continuous canola providing a viral “priming” function that results in an increase in viral load and species diversity [52]. It is possible, though unproven, that such priming could be mediated by the selection of various strains of *O. brassicae* as a result of high frequency canola cropping strategies.

## Conclusions

We found that the effects of long-term monoculture in canola production on soil nutrient fluxes and root exudate profiles were largely dependent on field location and associated environmental factors. While rotation was consistently associated with changes in fungal community composition in root, rhizosphere and bulk soil the effect on bacterial community composition was smaller and site-specific. Monoculture canola was consistently associated with known canola pathogens at all sites including *Alternaria, Leptosphaeria,* and *Phaeomycocentrospora* while soil from canola rotations that included wheat or barley were associated with higher levels of known cereal pathogens. Canola rotations were also associated with higher levels of potentially beneficial taxa including *Pseudomonas*, *Serratia*, *Penicillium* and *Chaetothyriaceae,* which have been previously linked to higher canola yield and were significantly associated with C-P-B rotations at multiple sites. The fungus *O. brassicae* was found to be both ubiquitous and highly abundant at all sites, with one specific strain strongly associated with monoculture canola at Lacombe.

By working in this model long-term rotation experiment, we were be able to measure important functional responses of the soil and plant microbiome to different canola frequencies over the past decade. Agricultural management practices such as crop rotation impose important changes on soil characteristics that accumulate over time. Identifying the positive and negative implications of canola frequency can be beneficial for refining the development of agronomic recommendations for canola production.

## Supplementary Information

**Additional file 1.** qPCR primer and probe sequences.

**Additional file 2.** Plant Root Simulator probe data.

**Additional file 3.** Agronomic data for each site and year.

**Additional file 4.** Bacterial and Fungal microbiome PERMANOVA.

**Additional file 5.** PCA Root exduate PERMANOVA.

**Additional file 6.** Indicator taxa analysis for each site and location. Includes specificity, sensitivity, *p* value, and taxonomic classification.

**Additional file 7.** Correlation valuesbetween abudance of *O. brassicae* ITS sequences and yield.

**Additional file 8.** Differential abundances of OTU determined using ALDEx2, including effect size, *p* value, and taxonomic classification.

**Additional file 9.** Root exudate amounts in root samples from each site, rotation, and year.

**Additional file 10.** Metadata for samples profiled using 16S rRNA gene sequencing.

**Additional file 11.** Metadata for samples profiled using ITS sequencing.

**Additional file 12.** ASV frequency table of 16S rRNA gene sequences for each sample.

**Additional file 13.** ASV frequency table of ITS sequences for each sample.

**Additional file 14.** Taxonomy of each 16S rRNA gene ASV.

**Additional file 15.** Taxonomy of each ITS ASV.

## Data Availability

Sequence files generated during the current study are available in the National Center for Biotechnology Information Short Read Archive under BioProject PRJNA892135 (https://www.ncbi.nlm.nih.gov/sra/PRJNA892135). Root exudate data for all samples, sites, and sampling years has been provided as Additional file 9. Metadata, the unrarefied OTU table, and corresponding taxonomic classifications have all been provided as Additional files 10, 11, 12, 13, 14 and 15.
