# Supplementary material for: Crop rotation significantly influences the composition of soil, rhizosphere, and root microbiota in canola (Brassica napus L.)
Source: Environ Microbiome. 2023 May 9;18:40. doi: 10.1186/s40793-023-00495-9 (PMC10169384; doi:10.1186/s40793-023-00495-9)
Supplement: Supplementary file 4 — Additional file 4. Bacterial and Fungal microbiome PERMANOVA. [file 40793_2023_495_MOESM4_ESM.docx]

|  |  |  | Scott | | | Swift | | | Lacombe | | |
| --- | --- | --- | --- | --- | --- | --- | --- | --- | --- | --- | --- |
|  |  |  | R^2^ | F | p | R^2^ | F | p | R^2^ | F | p |
| 16S | Soil | Year | 0.043 | 2.11 | 0.015 | 0.120 | 6.47 | 0.001 | 0.110 | 4.14 | 0.001 |
|  |  | Rotation | 0.103 | 2.54 | 0.002 | 0.072 | 1.94 | 0.004 | 0.089 | 1.68 | 0.020 |
|  |  | Cultivar | 0.019 | 0.94 | 0.457 | 0.014 | 0.78 | 0.790 | 0.030 | 1.14 | 0.241 |
|  | Rhizosphere | Year | 0.056 | 2.77 | 0.001 | 0.236 | 14.76 | 0.001 | 0.098 | 4.73 | 0.001 |
|  |  | Rotation | 0.049 | 1.21 | 0.105 | 0.062 | 1.94 | 0.010 | 0.064 | 1.55 | 0.001 |
|  |  | Cultivar | 0.023 | 1.13 | 0.253 | 0.013 | 0.83 | 0.604 | 0.027 | 1.32 | 0.080 |
|  | Root | Year | 0.144 | 7.93 | 0.001 | 0.201 | 11.90 | 0.001 | 0.168 | 8.94 | 0.001 |
|  |  | Rotation | 0.070 | 1.94 | 0.002 | 0.058 | 1.71 | 0.016 | 0.062 | 1.66 | 0.021 |
|  |  | Cultivar | 0.023 | 1.28 | 0.168 | 0.017 | 0.98 | 0.434 | 0.036 | 1.90 | 0.033 |
| ITS | Soil | Year | 0.164 | 9.99 | 0.001 | 0.268 | 18.16 | 0.001 | 0.137 | 10.32 | 0.001 |
|  |  | Rotation | 0.114 | 3.48 | 0.002 | 0.080 | 2.73 | 0.003 | 0.273 | 10.27 | 0.001 |
|  |  | Cultivar | 0.018 | 1.09 | 0.301 | 0.019 | 1.28 | 0.201 | 0.018 | 1.33 | 0.244 |
|  | Rhizosphere | Year | 0.098 | 5.50 | 0.002 | 0.362 | 30.34 | 0.001 | 0.031 | 2.13 | 0.085 |
|  |  | Rotation | 0.120 | 3.37 | 0.007 | 0.111 | 4.64 | 0.001 | 0.358 | 12.43 | 0.001 |
|  |  | Cultivar | 0.015 | 0.86 | 0.448 | 0.015 | 1.29 | 0.228 | 0.022 | 1.54 | 0.190 |
|  | Root | Year | 0.046 | 2.53 | 0.054 | 0.068 | 3.85 | 0.027 | 0.045 | 4.54 | 0.025 |
|  |  | Rotation | 0.151 | 4.14 | 0.002 | 0.189 | 5.33 | 0.002 | 0.501 | 25.04 | 0.001 |
|  |  | Cultivar | 0.016 | 0.89 | 0.426 | 0.035 | 1.98 | 0.112 | 0.024 | 2.38 | 0.115 |

Supplemental Table 5: Effect of experimental factors on the Bray-Curtis dissimilarity index for the bacterial (16S) and fungal (ITS) communities in the soil, root and rhizosphere Swift Current, Scott and Lacombe. Differences in microbiome composition in the soil, rhizosphere and root were compared using PERMANOVA.
