# Supplementary material for: Crop rotation significantly influences the composition of soil, rhizosphere, and root microbiota in canola (Brassica napus L.)
Source: Environ Microbiome. 2023 May 9;18:40. doi: 10.1186/s40793-023-00495-9 (PMC10169384; doi:10.1186/s40793-023-00495-9)
Supplement: Supplementary file 5 — Additional file 5. PCA Root exduate PERMANOVA. [file 40793_2023_495_MOESM5_ESM.docx]

|  |  | Df | SumsOfSqs | MeanSqs | F.Model | R^2^ | p value |
| --- | --- | --- | --- | --- | --- | --- | --- |
| Swift Current | Rotation | 2 | 2.04 | 1.02 | 0.17 | 0.02 | 0.999 |
|  | Cultivar | 1 | 3.02 | 3.02 | 0.52 | 0.03 | 0.733 |
|  | Rotation:Cultivar | 2 | 4.83 | 2.41 | 0.41 | 0.04 | 0.923 |
|  |  |  |  |  |  |  |  |
| Scott | Rotation | 2 | 9.07 | 4.53 | 0.87 | 0.09 | 0.539 |
|  | Cultivar | 1 | 1.78 | 1.78 | 0.34 | 0.02 | 0.869 |
|  | Rotation:Cultivar | 2 | 10.68 | 5.34 | 1.02 | 0.10 | 0.398 |
|  |  |  |  |  |  |  |  |
| Lacombe | Rotation | 2 | 37.00 | 18.50 | 5.02 | 0.32 | 0.002 |
|  | Cultivar | 1 | 2.69 | 2.69 | 0.73 | 0.02 | 0.518 |
|  | Rotation:Cultivar | 2 | 9.02 | 4.51 | 1.23 | 0.08 | 0.292 |

Supplemental Table 4: PERMANOVA of Principal Component Analysis (PCA) of the root exudate profiles from C-C, C-W and C-P-B canola at Swift Current, Scott and Lacombe.
